# An Efficient Method for the Differentiation of Human iPSC-Derived Endoderm toward Enterocytes and Hepatocytes

**DOI:** 10.3390/cells10040812

**Published:** 2021-04-06

**Authors:** Shimeng Qiu, Yaling Li, Yuki Imakura, Shinji Mima, Tadahiro Hashita, Takahiro Iwao, Tamihide Matsunaga

**Affiliations:** 1 Department of Clinical Pharmacy, Graduate School of Pharmaceutical Sciences, Nagoya City University, 3-1 Tanabe-dori, Mizuho-ku, Nagoya 467-8603, Japan; kaeffie9325@hotmail.com (S.Q.); liyaling6868@gmail.com (Y.L.); thashita@phar.nagoya-cu.ac.jp (T.H.); tiwao@phar.nagoya-cu.ac.jp (T.I.); 2 Bio Science & Engineering Laboratory, FUJIFILM Corporation, 577, Ushijima, Kaisei-machi, Ashigarakami-gun, Kanagawa 258-8577, Japan; yuki.imakura@fujifilm.com (Y.I.); shinji.mima@fujifilm.com (S.M.)

**Keywords:** human induced pluripotent stem cell, endoderm, enterocyte, hepatocyte, drug development, pharmacokinetics study, differentiation

## Abstract

The endoderm, differentiated from human induced pluripotent stem cells (iPSCs), can differentiate into the small intestine and liver, which are vital for drug absorption and metabolism. The development of human iPSC-derived enterocytes (HiEnts) and hepatocytes (HiHeps) has been reported. However, pharmacokinetic function-deficiency of these cells remains to be elucidated. Here, we aimed to develop an efficient differentiation method to induce endoderm formation from human iPSCs. Cells treated with activin A for 168 h expressed higher levels of endodermal genes than those treated for 72 h. Using activin A (days 0–7), CHIR99021 and PI−103 (days 0–2), and FGF2 (days 3–7), the hiPSC-derived endoderm (HiEnd) showed 97.97% CD−117 and CD−184 double-positive cells. Moreover, HiEnts derived from the human iPSC line Windy had similar or higher expression of small intestine-specific genes than adult human small intestine. Activities of the drug transporter P-glycoprotein and drug-metabolizing enzyme cytochrome P450 (CYP) 3A4/5 were confirmed. Additionally, Windy-derived HiHeps expressed higher levels of hepatocyte- and pharmacokinetics-related genes and proteins and showed higher CYP3A4/5 activity than those derived through the conventional differentiation method. Thus, using this novel method, the differentiated HiEnts and HiHeps with pharmacokinetic functions could be used for drug development.

## 1. Introduction

For pathological research, drug development, and organ transplant, obtaining healthy human organs, tissues, and cells is challenging. The discovery of human induced pluripotent stem cells (iPSCs), which can be differentiated into any cell type, has brought hope to individuals with severe illnesses [1]. Human iPSCs can differentiate into embryonic germ layers that comprise the endoderm, ectoderm, and mesoderm, which are present in most animal embryos during gastrulation [2]. Subsequently, the three germ layers differentiate into specific tissues or organs.

Drug efficacy, safety, and pharmacokinetics strongly influence the success rate of new drugs [3,4]. Orally administrated drugs pass via the gastrointestinal tract and are mainly absorbed in the small intestine. Many oral drugs are also metabolized in the small intestine [5,6]. Then, the drugs enter systemic circulation after they are metabolized in the liver [7]. In vivo and in vitro models have been developed to evaluate the efficiency, safety, and pharmacokinetics of a drug. However, because of inter-species differences, experimental animals cannot accurately evaluate drug efficiency, safety, and pharmacokinetics [8,9]. Caucasian colon adenocarcinoma (Caco−2) cells have been used to evaluate oral drug absorption in the small intestine, although expression patterns of drug transporters in Caco−2 cells differ from those of the small intestine in humans [8,9,10]. For predicting drug metabolism in the small intestine, small intestinal microsomes are widely used; however, microsomes can only evaluate drug metabolism via enzymes present in the fraction. Human primary hepatocytes and liver microsomes are used for drug metabolism and uptake assessment [11]. However, obtaining a stable supply of the same batch of hepatocytes, which maintain a high level of liver function, is challenging [12]. Human iPSC-derived enterocytes that express both drug transporters and drug-metabolizing enzymes have been reported [13,14,15,16]. Moreover, human iPSC-derived hepatocytes (HiHeps) with pharmacokinetic-related functions have been reported [13,14,15]. However, generation of more mature enterocytes or hepatocytes from human iPSCs has not been reported. Therefore, we focused on promoting the human iPSC-derived endoderm (HiEnd) differentiation efficiency to optimize enterocyte and hepatocyte differentiation because both are derived from the endoderm [16,17,18].

Various methods of endoderm differentiation from human iPSCs have been reported [18,19,20]. However, most studies have used human iPSCs maintained under on-feeder coculture conditions. The use of human iPSCs (on-feeder) may lead to contamination from other cell lineages and fluctuation of differentiation efficiency between human iPSC lines [21,22]. Thus, using human iPSCs maintained under feeder-free conditions to differentiate target-cells or tissues is desirable. Hence, whether the applicability of the protocol used in human iPSCs (on feeders) could be adapted to human iPSCs (feeder-free) should be discussed. We failed to differentiate human iPSCs (feeder-free) into endoderm using the same protocol, which we earlier reported for the differentiation of human iPSCs (on-feeder) into endoderm. Different human iPSC culture conditions result in differentiation efficiency variations, even when using the same human iPSC line. Furthermore, different pluripotent cell lines exhibit variations in differentiation efficiency [23]. Therefore, developing a method that is adaptable to human iPSC lines is warranted.

To gain a high-quality endoderm, we developed an efficient method to differentiate human iPSCs (feeder-free) into the endoderm based on a previous report [22,24]. Moreover, we used 1% dimethyl sulfoxide (DMSO) for 24 h pretreatment before differentiation and increased the differentiation period from 3 or 5 to 7 days. We confirmed the applicability of this method using three types of human iPSC lines. Then, we evaluated the differentiated endoderm for the presence of CD117- and CD184-, which are endodermal markers, double-positive cells through flow cytometry [25]. Further, we differentiated the endoderm into enterocytes and hepatocytes and evaluated expression levels of specific genes and proteins and pharmacokinetics-related functions.

## 2. Materials and Methods

### 2.1. Human iPSCs

Human iPSC lines Windy and K were provided by Umezawa et al. at the National Center for Child Health and Development. The human iPSC line FF−2 was provided by Fujifilm Corporation (Tokyo, Japan). On-feeder culture: Windy was cultured on mitomycin C (Kyowa Kirin Co., Tokyo, Japan)-treated mouse embryonic fibroblasts from embryonic day 14. The culture medium was consist of DMEM/F−12 (Wako Pure Chemical Industries, Osaka, Japan) supplemented with 20% KnockOut Serum Replacement (Thermo Fisher Scientific, Carlsbad, CA, USA), 2 mM L-glutamine (Wako Pure Chemical Industries), 1% minimum essential medium nonessential amino acid solution (NEAA) (Wako Pure Chemical Industries), 0.1 mM 2-mercaptoethanol (β-MeE) (Sigma-Aldrich, St. Louis, MO, USA), and 5 ng/mL basic fibroblast growth factor (FGF2) (PeproTech Inc., Rocky Hill, NJ, USA). Feeder-free culture: Windy and FF−2 were maintained using mTeSR1 (Veritas Corporation, Tokyo, Japan) on growth factor-reduced Matrigel (BD Biosciences, Bedford, MA, USA)-coated plates. K was cultured using STEMUP (Nissan Chemical Corporation, Tokyo, Japan). Furthermore, 0.2 µg/cm^2^ iMatrix−511 silk (Matrixome Inc., Osaka, Japan)-coated plates were used for cell maintenance. Before differentiation, K was subcultured in 0.5 µg/cm^2^ iMatrix−511 silk-coated plates.

### 2.2. HiEnd Differentiation

Human iPSCs cultured under on-feeder conditions were differentiated into the endoderm based on a previously reported protocol [26]. In brief, when human iPSCs grew at a confluency of 70%, differentiation was initiated using activin A (PeproTech Inc.) for 3, 5 or 7 days. The concentration of fetal bovine serum (FBS) (Sigma-Aldrich,) in the basal medium was maintained at 0.5% for 2 days. Then, it was increased to 2%.

Human iPSCs (feeder-free) were differentiated using three protocols, namely A (Activin A), ACP (Activin A, CHIR99021 and PI−103), and ABF (Activin A, BMP4 and FGF2). Protocol A was reported [27]. Briefly, cells were treated with 100 ng/mL activin A for 7 d. From day 1 to day 7, bone morphogenetic protein−4 (BMP4) (R&D systems, Inc., Minneapolis, MN, USA), vascular endothelial growth factor (R&D systems, Inc.), and FGF2 were added. When the confluency of human iPSCs reached nearly 90%, the ACP protocol was used by adding the basal medium RPMI 1640 + GlutaMAX medium (Thermo Fisher Scientific), containing 100 units/mL penicillin G, 100 µg/mL streptomycin sulfate (Cosmo Bio Co., Tokyo, Japan), 1% NEAA, 0.2× B27 supplement minus vitamin A (Thermo Fisher Scientific), 100 ng/mL activin A, 2 µM CHIR99021 (Focus Biomolecules, Plymouth Meeting, PA, USA), and 50 nM PI−103 (ChemScene LLC, Monmouth Junction, NJ, USA) to the cells and culturing them for 48 h. Then, the same basal medium, containing 100 units/mL penicillin G, 100 µg/mL streptomycin sulfate, 1% NEAA, 0.2× B27 supplement minus vitamin A, 100 ng/mL activin A, and 20 ng/mL FGF2 were used to culture cells for 24 h. Thereafter, RPMI 1640 + GlutaMAX medium was changed to a mixture containing Advanced RPMI1640 medium (Thermo Fisher Scientific): RPMI 1640 + GlutaMAX medium = 1:3, containing 100 units/mL penicillin G, 100 µg/mL streptomycin sulfate, 1% NEAA, 0.2× B27 supplement minus vitamin A, 100 ng/mL activin A, and 20 ng/mL FGF2 to culture cells for 96 h. The human iPSCs were differentiated using the ABF protocol when they reached a confluency of 90%. Cells were treated by supplementation of the basal medium RPMI 1640 + GlutaMAX medium (Thermo Fisher Scientific), which contained 100 units/mL penicillin G, 100 µg/mL streptomycin sulfate (Cosmo Bio Co.), 1% NEAA, 0.2× B27 supplement minus vitamin A (Thermo Fisher Scientific), 100 ng/mL activin A for 5 days, 10 ng/mL BMP4 and 20 ng/mL FGF2 were only added for the initial 24 h.

### 2.3. Differentiation of Small Intestinal Stem Cells

The endoderm induced from human iPSCs (on-feeder culture) was cultured in DMEM/F12, 2% FBS, 1% GlutaMAX (Thermo Fisher Scientific), 100 units/mL penicillin G, 100 µg/mL streptomycin sulfate, and 250 ng/mL FGF2 for 4 days [28]. Regarding human iPSCs (feeder-free culture), the endoderm was differentiated to small intestinal stem cells using DMEM/F12 containing 2% FBS, 2× B27 supplement (Thermo Fisher Scientific), 1% GlutaMAX, 100 units/mL penicillin G, 100 µg/mL streptomycin sulfate, and 250 ng/mL FGF2.

### 2.4. Small Intestinal Stem Cell-Derived Enterocyte Differentiation

The enterocytes were differentiated as reported [27]. Briefly, human iPSC-derived small intestinal stem cells were subcultured in 24- or 96-well plates or 24-well inserts and cultured using enterocyte maturation medium and 10 μM Y−27632 (Focus Biomolecules) for 24 h. The enterocyte maturation medium consisted of DMEM/F12 containing 2% FBS, 2× B27 supplement, 1× N2 supplement, 1% NEAA, 100 units/mL penicillin G, 100 µg/mL streptomycin sulfate, 1% GlutaMax, and 50 ng/mL epidermal growth factor (PeproTech Inc.). Then, cells were cultured in enterocyte maturation media supplemented with 30 μM forskolin for 18 days. From the seventh day of the 18-days culture, PD98059 (Wako Pure Chemical Industries), 5 µM 5-aza−2′-deoxycytidine (5-aza−2′-dC) (Wako Pure Chemical Industries), and 0.5 µM A−83–01 (Wako Pure Chemical Industries) were added to mature the human iPSC-derived enterocyte (HiEnt).

### 2.5. Hepatic Differentiation

Human iPSCs (on-feeder culture) were differentiated into hepatocytes based on our study [14]. Briefly, human iPSCs were treated with activin A for 5 days. Then, differentiated cells (1 × 10^5^ cells/cm^2^) were subcultured onto Matrigel-coated 24- or 96-well plates. Human iPSCs cultured without feeder cells were differentiated into endodermal cells using the ACP protocol. Endodermal cells (2 × 10^5^ cells/cm^2^) were passaged onto Matrigel-coated 24- or 96-well plates. The subcultured cells were first cultured in the presence of 1% DMSO for 7 days, and then Cosmedium 004 (Cosmo Bio Co.) supplemented with 10 ng/mL hepatocyte growth factor (PeproTech, Inc.), 20 ng/mL oncostatin M (Wako Pure Chemical Industries), 100 nM dexamethasone (Wako Pure Chemical Industries), and 2 mM valproic acid (Wako Pure Chemical Industries) for 7 days. During the last 3 days, 25 μM celecoxib (Tokyo Chemical Industry Co. Ltd., Tokyo, Japan) was added to the culture. The medium was then changed to Cosmedium 004 supplemented with 10 ng/mL hepatocyte growth factor, 20 ng/mL oncostatin M, 100 nM dexamethasone, and 25 μM celecoxib for 3 days. Finally, cells were cultured in Cosmedium 004 containing 25 μM celecoxib for 4 days.

The catalog number of reagents used for cell differentiation were summarized in Table S1.

### 2.6. Real-Time RT-PCR

Total cellular RNA was extracted using the Agencourt RNAdvance Tissue Kit (Beckman Coulter, Brea, CA, USA) as per the manufacturer’s instructions. We then synthesized cDNA for real-time PCR from 0.8 ng total RNA using the ReverTra Ace qPCR RT Master Mix (Toyobo, Osaka, Japan) as per the manufacturer’s instructions. Real-time PCR was performed using the KAPA SYBR FAST qPCR Master mix (2×) Kit ABI Prism (Sigma-Aldrich) and detected using LightCycler^®^ 96 System (Roche Diagnostics, Basel, Switzerland). Primer sequences for PCR are listed in Table 1. Gene expression was quantified using the 2^−ΔΔCT^ method and normalized to that of hypoxanthine phosphoribosyltransferase (HPRT).

### 2.7. Immunofluorescence Staining

The cells were fixed and permeabilized using methanol (−20 °C) for CDX2 staining. For zonula occludens−1 (ZO−1), villin, mucin2 (Muc2), P-glycoprotein (P-gp), albumin, multidrug resistance-associated protein 2 (MRP2), and alpha fetoprotein (AFP) staining, 4% paraformaldehyde (Wako Pure Chemical Industries) was used to fix the cells. Permeabilization was then conducted using 0.1% Triton X−100 for 5 min. Next, the cells were blocked with 5% FBS in phosphate-buffered saline (PBS). The cells for P-gp staining were blocked using 0.1% bovine serum albumin (Thermo Fisher Scientific) at each wash step. The cells were incubated with primary antibodies overnight at 4 °C, and subsequently with secondary antibodies for 60 min at 25 °C. The primary and secondary antibodies used in the immunofluorescence staining are summarized in Table 2.

Then, cells were incubated with 0.2 µg/mL 4′,6-diamidino−2-phenylindole (DAPI) (Dojindo Molecular Technologies, Inc., Kumamoto, Japan) for 5 min to visualize nuclei. The stained cells were viewed using the All-in-One Fluorescence Microscope BZ-X800 (Keyence, Osaka, Japan).

### 2.8. Flow Cytometry

HiEnd was dissociated using TrypLE^TM^ Select (Thermo Fisher Scientific). The number of cells was adjusted to between 1 × 10^5^ and 1 × 10^6^/mL. LIVE/DEAD™ Fixable Aqua Dead Cell Stain Kit (Thermo Fisher Scientific) was used to differentiate between live and dead cells. Then, cells were fixed in 4% paraformaldehyde for 15 min at 25 °C and preserved (for no longer than 1 week) in FACS buffer (PBS with 2% FBS) for CD184 and CD117 staining using anti-human CD184 (BioLegend, San Diego, CA, USA) and CD117 antibody (Thermo Fisher Scientific). Finally, stained cells were analyzed using Attune NxT Flow Cytometer (Thermo Fisher Scientific), and the percentages of double-positive cells were calculated by comparing with the cells stained using IgG1 (BD Biosciences) and IgG2a (Thermo Fisher Scientific), which were used as isotype controls.

### 2.9. Bidirectional Transporter Assay

HiEnts cultured on cell culture inserts were used for the bidirectional transporter assay. After maturation, cells were washed with the transporter buffer containing Hank’s balanced salt solution (HBSS) (Thermo Fisher Scientific) supplemented with 10 mM 4-(2-hydroxyethyl) −1-piperazineethanesulfonic acid (HEPES) (Thermo Fisher Scientific). Rhodamine 123 (Sigma-Aldrich) and verapamil (Wako Pure Chemical Industries) were used as the substrate and inhibitor of P-gp, respectively. The cells were preincubated with transporter buffer in the presence or absence of 100 μM verapamil for 20 min at 37 °C. After preincubation, the buffer in the apical or basal chamber was changed to rhodamine 123-containing transporter buffer with or without verapamil for 45 min at 37 °C. Then, the solution in the receiver chamber was collected and the concentration of rhodamine 123 was quantified using Synergy HTX (BioTek, Winooski, VT, USA), with the excitation, and emission wavelengths of rhodamine 123 being 485 and 535 nm, respectively.

### 2.10. Calculations

For apical-to-basal transport, we calculated values of apparent permeability coefficient *(P*_app_) and efflux ratio (ER) of rhodamine 123 based on a previously reported analytical method [29].

### 2.11. Activity of Drug-Metabolizing Enzymes

Differentiated cells were washed using PBS and incubated with HBSS, containing 10 µM midazolam (Wako Pure Chemical Industries), which is a substrate for cytochrome P450 (CYP) 3A4/5, in the presence or absence of 10 µM ketoconazole (a CYP3A4/5 inhibitor) (Tokyo Chemical Industry Co. Ltd.) at 37 °C. The assay was stopped by placing the cell culture plate on ice and the supernatant was collected for analyzing the metabolite. Cellular protein in each well was quantified using Pierce™ BCA Protein Assay Kit (Thermo Fisher Scientific) and used for normalizing metabolic activity. The metabolite 1-hydroxymidazolam was analyzed through ultraperformance liquid chromatography-tandem mass spectrometry (UPLC-MS/MS) using QTRAP^®^ 6500 (AB SCIEX, Tokyo, Japan) and Nexera XR (Shimadzu, Kyoto, Japan) equipped with the ACQUITY UPLC BEH C18 column (2.1 × 50 mm, 1.7 μm) (Waters corporation, Milford, MA, USA). Mobile phases included ultrapure water (A) and methanol (B) containing 0.1% formic acid. Temperatures of the column and samples were set at 40 °C and 4 °C, respectively. Gradient conditions of the mobile phase were 0–0.5 min: 30% B, 0.5–2.0 min: 30%→50% B, 2.0–3.5 min: 50%→80% B, 3.5–3.6 min: 80%→95% B, 3.6–4.6 min: 95% B, 4.6–4.8 min: 95%→30% B, and 4.8–6.0 min: 30% B; the flow rate was 0.3 mL/min. For MS, curtain gas; 20 psi, nebulizer gas; 25 psi, turbo gas; 60 psi, ion spray voltage; 2000 V, and temperature; 350 °C were used. The multiple reaction monitoring mode was used to detect 1′-hydroxymidazolam (*m*/*z* 341.904→324.000) and chlorpropamide (*m*/*z* 277.009→111.000). The retention time was 2.0 min for 1′-hydroxymidazolam and 3.3 min for chlorpropamide (internal standard). Data analysis was performed using the MultiQuant software. Calibration curves were prepared using 0.1–10 ng/mL 1′-hydroxymidazolam.

### 2.12. Statistical Analysis

Statistical significance was assessed using Student’s *t*-test to compare two groups or analysis of variance followed by Tukey’s test for multiple comparisons.

## 3. Results

### 3.1. Evaluation of Differentiation Method with Respect to iPSC-Derived Endoderm, Small Intestinal Stem Cells, and Enterocytes

We optimized the iPSC-derived endoderm differentiation protocol. Moreover, we used the differentiated endoderm for the further differentiation into small intestinal stem cells and enterocytes.

#### 3.1.1. Verification of 72 h Activin a Treatment in Human iPSC (Feeder-Free) Differentiation into the Endoderm

Compared with human iPSCs growing under feeder-free conditions, those maintained on-feeder cells exhibited lower proliferation rates, contamination from other animal cells, and operational complication. We differentiated human iPSCs (feeder-free) using the 72 h activin A differentiation method we reported for human iPSCs (on-feeder) [28] (Figure S1a). We confirmed the morphologies of undifferentiated and differentiated cells and found that the density of differentiated cells was low (Figure S1b). We evaluated the efficiency of endoderm differentiation through gene expression levels of octamer-binding transcription factor 4 (OCT4), which is an undifferentiated marker, and forkhead-box protein A1 (FOXA1), SRY-box 17 (SOX17), and GATA-binding protein 4 (GATA4), which are endoderm markers. The human iPSC (on-feeder)-derived endoderm exhibited significantly higher expression levels of endoderm-specific genes than the human iPSC (feeder-free)-derived endoderm. Levels of FOXA1, SOX17, and GATA4 expression in the human iPSC (on-feeder)-derived endoderm were 96-, 107- and 1022-fold higher than those of human iPSCs, respectively (Figure S1c). Therefore, it was difficult to differentiate human iPSC (feeder-free)-derived endoderm using the 72 h activin A treatment method.

#### 3.1.2. Duration of Activin a Treatment

**Figure 1:**
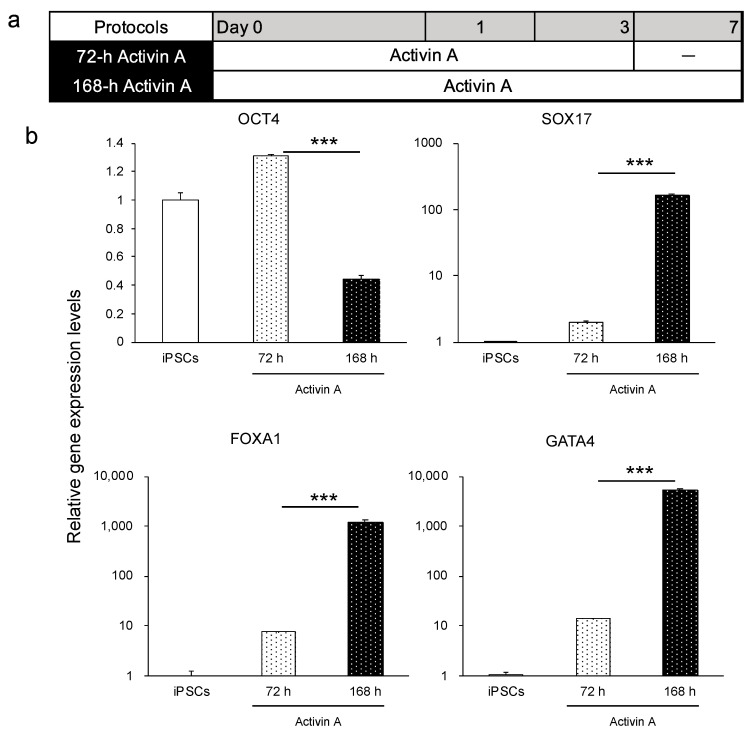
Comparison of duration of activin A treatment (72 and 168 h) on the differentiation of the iPSC line Windy (feeder-free) into the endoderm. (**a**) Human iPSC-derived endoderm differentiation protocols, namely 72 h activin A and 168 h activin A. (**b**) Relative gene expression levels of OCT4, SOX17, FOXA1, and GATA4. Human iPSCs = 1. Results are presented as means ± S.D. (*n* = 3). Levels of statistical significance: *** *p* < 0.001.

Activin A, a member of the transforming growth factor-β family, is used as a determinant of endodermal differentiation [30,31,32]. Considering that the duration of activin A treatment might influence the efficiency of endoderm differentiation, we compared 72 h (3 days) and 168 h (7 days) of activin A treatment using the human iPSC line Windy (feeder-free) (Figure 1a). Compared with the 72 h treatment group, the 168 h treatment group expressed higher levels of endoderm-specific genes, such as *SOX17*, *FOXA1*, and *GATA4*, and lower levels of *OCT4*, under the feeder-free culture condition (Figure 1b). Thus, the duration of activin A treatment influences the efficiency of endoderm differentiation. Under on-feeder culture conditions, the cells could not survive until day 7 (data not shown). Confirming that the 168 h treatment of activin A increased the efficacy of human iPSCs (feeder-free) to undergo endoderm differentiation, we extended the duration of activin A treatment to 7 days.

#### 3.1.3. Investigation of Differentiation Method to the Anterior Primitive Streak

**Figure 2:**
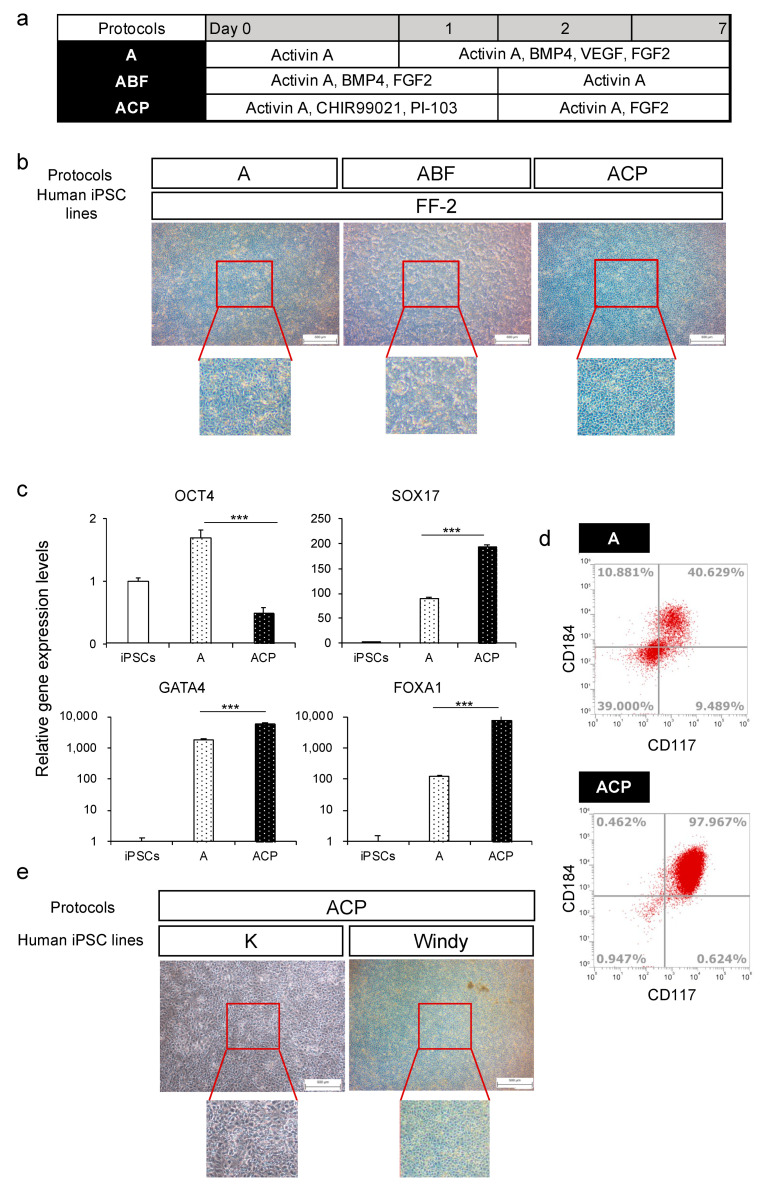
A comparison of protocols A, ABF, and ACP on human iPSC (feeder-free) differentiation into the endoderm. (**a**) Human iPSC-derived endoderm (HiEnd) differentiation using protocols A, ABF, and ACP. (**b**) Morphology of human iPSC line FF−2-derived HiEnd using protocols A, ABF, and ACP. (**c**) Relative gene expression levels of undifferentiated cell markers and endodermal markers in the FF−2-derived endoderm using protocols A and ACP. (**d**) Flow cytometric analysis of CD184 and CD117 in FF−2-derived endoderm using protocols A and ACP. (**e**) Morphology of human iPSC line K and Windy-derived-HiEnd using the ACP protocol. Results are presented as means ± S.D. (*n* = 3). Human iPSCs = 1. Scale bars = 500 μm. Levels of statistical significance: *** *p* < 0.001.

Human iPSCs differentiate into the endoderm through the anterior primitive streak (APS) [31], and several differentiation methods of APS from human iPSCs have been reported [31,33]. We compared three differentiation methods of APS to verify which was best for endoderm differentiation using the human iPSC line FF−2 (Figure 2a). At the end of endoderm differentiation, we observed the morphologies of differentiated endoderm. The results revealed that the ABF group had a relatively heterogeneous population of cells, whereas groups A and ACP exhibited a relatively homogeneous and flatter population of cells (Figure 2b). Then, we analyzed the OCT4, SOX17, FOXA1, and GATA4 expression levels of groups A and ACP. Although group A had higher expression levels of endoderm markers, such as SOX17, GATA4, and FOXA1, expression level of the undifferentiated marker OCT4 also increased compared with undifferentiated human iPSCs. The ACP group showed the highest expression levels of endoderm markers and lowest OCT4 expression level (Figure 2c). Furthermore, we evaluated CD117 and CD184 protein expression using flow cytometry. The rates of double-positive cells in group A varied between experiments from about 40% to 80%. By contrast, those in group ACP were always more than 95% (Figure 2d). Subsequently, we evaluated the applicability of protocol ACP using the other two human iPSC lines, K and Windy. Regarding cell morphology, we obtained a uniform cell population using the ACP protocol (Figure 2e). Thus, we used the ACP protocol for further investigation, because it was thought that the protocol would be useful for endodermal differentiation.

#### 3.1.4. Evaluation of the Effect of DMSO on the Endoderm, Small Intestinal Stem Cells, and Enterocyte Differentiation

**Figure 3:**
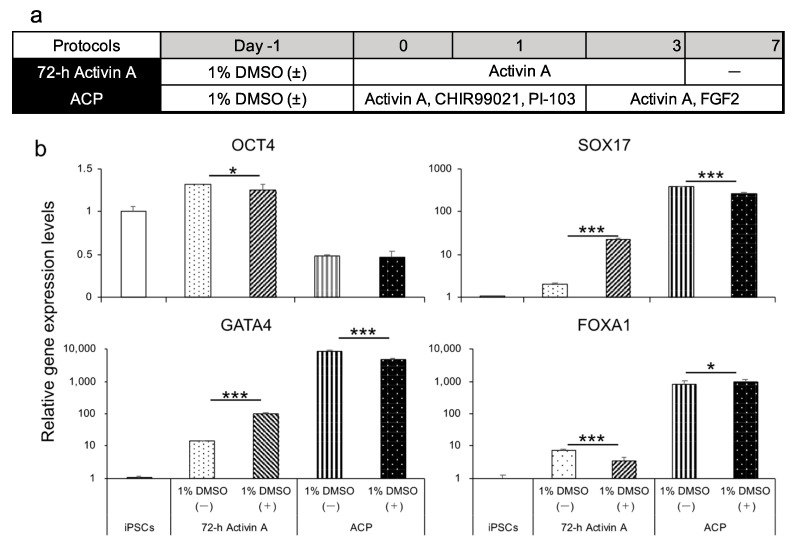
A comparison of differentiated endoderm on the pretreatment or absence of 1% DMSO using 72 h activin A and ACP protocols. (**a**) Human iPSC-derived endoderm differentiation method using 72 h activin A and ACP protocols in the presence or absence of 1% DMSO. (**b**) Relative gene expression levels of undifferentiated cell markers and endodermal markers in the human iPSC line Windy-derived endoderm using 72 h activin A and ACP protocols with or without pretreatment with 1% DMSO. Results are presented as means ± S.D. (*n* = 3). Human iPSCs = 1. Levels of statistical significance: * *p* < 0.05, *** *p* < 0.001.

Several reports have shown that DMSO induces cell differentiation toward three germ layers, summarized by Sambo et al. [34]. We attempted to differentiate human iPSCs (feeder-free) into the endoderm using protocol 72 h activin A and ACP with or without 1% DMSO, 24 h before differentiation (Figure 3a). The endoderm derived from Windy was analyzed for gene expression levels. We found that the endoderm differentiated using protocol 72 h activin A with 1% DMSO had higher SOX17 and GATA4 expression levels. Gene expression levels of the other markers decreased significantly (Figure 3b).

**Figure 4:**
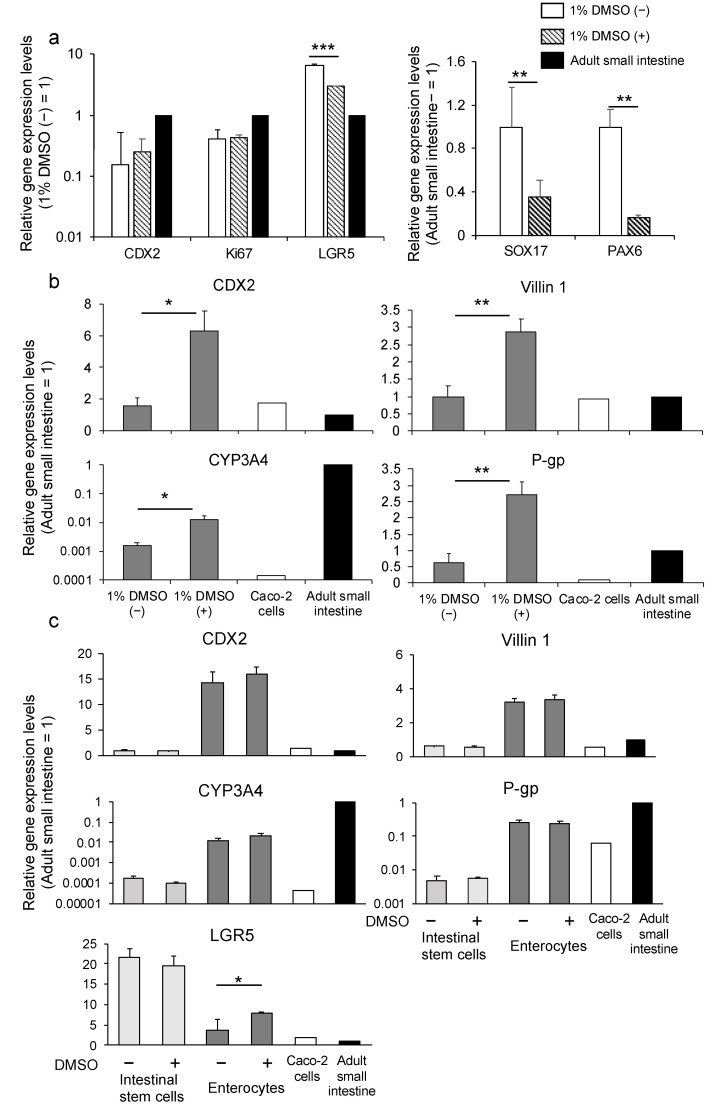
The effect of 1% DMSO pretreatment before differentiation on small intestinal differentiation. (**a**) Relative gene expression levels of CDX2, SOX17, Pax−6, Ki67, and LGR5 in human iPSC line K-derived small intestinal stem cells using the ACP protocol in the presence or absence of 1% DMSO. (**b**) Relative gene expression levels of CDX2, villin 1, CYP3A4, and P-gp in human iPSC line K-derived enterocytes using the ACP protocol with or without 1% DMSO. (**c**) Relative gene expression levels of CDX2, villin 1, CYP3A4, P-gp, and LGR5 in Windy-derived intestinal stem cells and enterocytes using the ACP protocol with or without 1% DMSO. Results are presented as means ± S.D. (*n* = 3). Human small intestine = 1. Levels of statistical significance: * *p* < 0.05, ** *p* < 0.01, *** *p* < 0.001.

We differentiated the endoderm toward small intestinal stem cells and enterocytes using the protocol we reported [27]. We evaluated gene expression levels in the small intestinal stem cells generated from the K-derived endoderm. We found that cells in the 1% DMSO pretreatment group (1% DMSO (+) group) showed a higher expression level of caudal-type homeobox 2 (CDX2), which is a hindgut marker [35], lower expression levels of SOX17 and paired box protein Pax−6 (PAX6), which is a neuroectoderm marker [36], and almost the same expression level of Ki67, which is a proliferation marker, compared with those of the group without 1% DMSO pretreatment (1% DMSO (–) group) (Figure 4a). Although the leucine-rich repeat-containing G protein-coupled receptor 5 (LGR5) expression level was suppressed in the 1% DMSO (+) group compared with the 1% DMSO (−) group, it was higher than that of the human adult small intestine (Figure 4a). To identify the effect of 1% DMSO on further differentiation, we induced small intestinal stem cells to differentiate into enterocytes according to the protocol we reported [27]. Gene expression levels of CDX2 and villin 1 (enterocyte-related markers), CYP3A4 (drug-metabolizing enzyme), and P-gp (drug transporter) were significantly higher in the 1% DMSO (+) group compared with that in the 1% DMSO (−) group (Figure 4b). Thus, we verified the effectiveness of DMSO pretreatment for differentiation into endoderm using cell line K. Moreover, we used another human iPSC line Windy to evaluate the ability of DMSO in endoderm to differentiate into enterocytes. Differentiated enterocytes showed higher or similar levels of LGR5, CDX2, and villin 1 and lower levels of P-gp and CYP3A4 expression, compared to the human adult small intestine. Although CYP3A4 expression level remained lower than that of human adult intestine, it was 450 times higher than that in Caco−2 cells. However, excluding LGR5, 1% DMSO pretreatment did not significantly influence target gene expression levels between differentiated cells and Windy (Figure 4c).

### 3.2. Differentiation into Enterocytes Using Endoderm Generated by Protocol ACP

**Figure 5:**
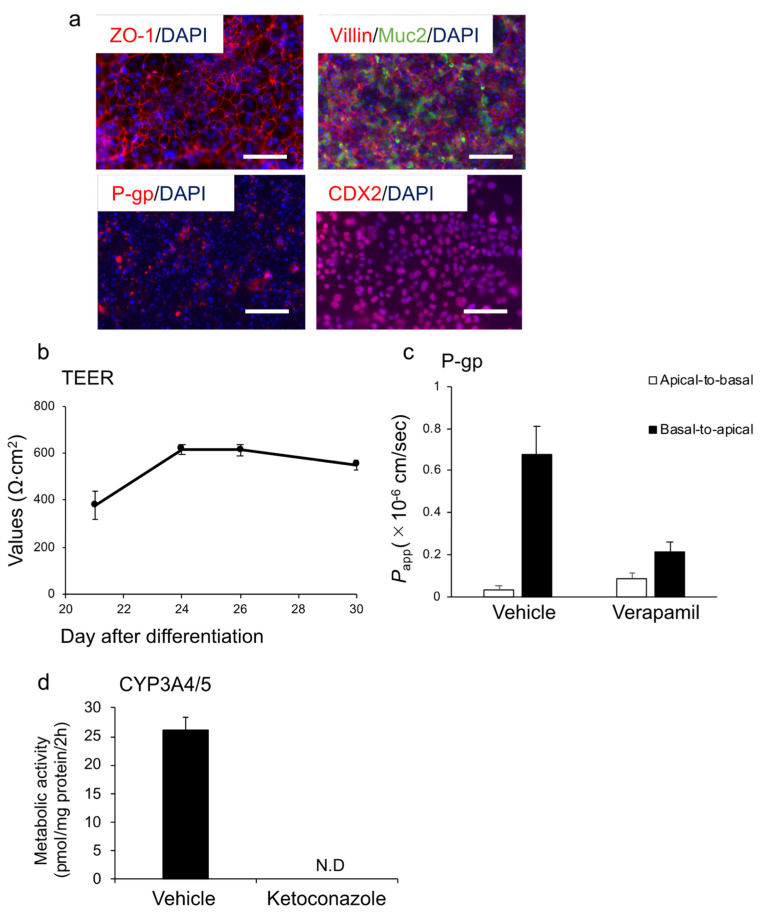
Enterocyte differentiation from the human iPSC line Windy (feeder-free)-derived endoderm using the ACP protocol. (**a**) Immunofluorescence images of ZO−1 (red), villin (red), Muc2 (green), P-gp (red), and CDX2 (red). Nuclei were stained with DAPI (blue). (**b**) TEER values of cells were measured from days 21 to 30. (**c**) Cells were incubated with the transport buffer containing rhodamine 123 at 37 °C for 45 min in the presence or absence of 100 μM verapamil, a P-gp inhibitor. (**d**) CYP3A4/5 activity in the presence or absence of 10 μM ketoconazole, a CYP3A4 inhibitor. All data are presented as mean ± S.D. (*n* = 3). Scale bars = 50 μm. N.D., not detected.

We further investigated the characteristics of enterocytes differentiated from HiEnd using the ACP protocol (ACP–enterocyte) using the Windy cell line. Protein expression of ZO−1 (tight junction marker) [37], villin, CDX2, Mucin 2 (goblet cell marker), and P-gp were confirmed in enterocytes using immunofluorescence staining (Figure 5a). The transepithelial electrical resistance (TEER) value, which is an index of the robustness of tight junctions, was measured from days 21 of differentiation to the end of differentiation. TEER values were stably maintained at 500–600 Ω·cm^2^ from days 24 to the end of differentiation (Figure 5b). Then, we analyzed the ability of the efflux transporter P-gp using rhodamine 123 as the substrate. The *P*_app_ values were 0.04 ± 0.02 and 0.09 ± 0.03 (×10^−6^ cm/s) in the absence and presence of verapamil, a specific P-gp inhibitor, respectively. Moreover, the basal-to-apical *P*_app_ values were 0.68 ± 0.13 and 0.21 ± 0.05 (×10^−6^ cm/s) in the absence and presence of verapamil, respectively. The ER in the vehicle group was 18.4, which decreased to 2.4 in the presence of verapamil (Figure 5c). We also quantified 1′-hydroxylmidazolam, which is a major metabolite of midazolam, to evaluate CYP3A4/5 activity and found that the cells possessed CYP3A4/5 activity. In the presence of ketoconazole, an inhibitor of CYP3A4/5, the activity was markedly suppressed (Figure 5d). According to the abovementioned results, we considered that the human iPSC-derived endoderm cells obtained using the ACP protocol exhibited the ability to differentiate into enterocytes.

### 3.3. Differentiation into Hepatocytes Using Endoderm Generated by Protocol ACP

**Figure 6:**
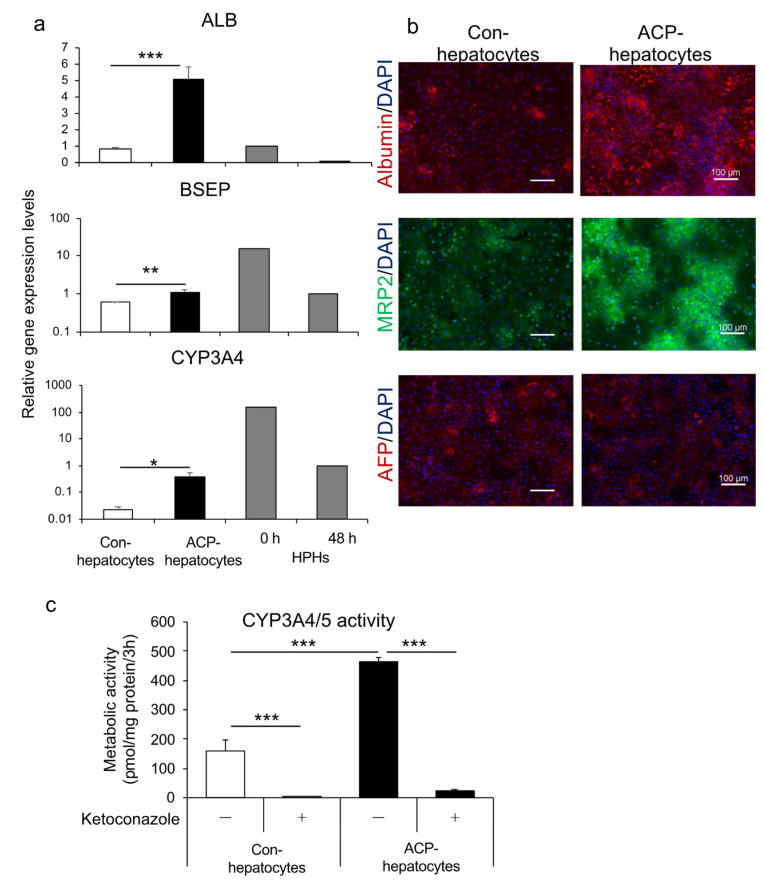
A comparison of the human iPSC line Windy cultured with or without feeder cell-derived endoderm during hepatocyte differentiation. (**a**) Relative gene expression of *ALB*, *BSEP*, and *CYP3A4*. The mRNAs of cryopreserved human primary hepatocytes were collected at 0 and 48 h of culture. (**b**) Immunofluorescence images of albumin (red), MRP2 (green), and AFP (red). Nuclei were stained with DAPI (blue). (**c**) CYP3A4/5 activity in the presence or absence of 10 μM ketoconazole, a CYP3A4 inhibitor. Scale bars = 100 μm. All data are presented as mean ± S.D. (*n* = 3). Levels of statistical significance: * *p* < 0.05, ** *p* < 0.01, *** *p* < 0.001.

We also differentiated the endoderm generated using the ACP protocol to hepatocytes (ACP-hepatocytes), according to the method reported by our group [14]. The differentiated hepatocytes showed higher gene expression levels of albumin (ALB), a hepatocyte marker, bile salt export pump (BSEP), and CYP3A4, significantly, compared with hepatocytes differentiated using conventional methods (con-hepatocytes), which we have reported for hepatocyte differentiation [14] (Figure 6a). Further, the expression of albumin and MRP2, a drug transporter, and similar levels of AFP, which is found in the fetal liver during development, were higher than those of con-hepatocytes (Figure 6b). Both types of hepatocytes exhibited CYP3A4/5 activity. With the addition of ketoconazole, the activity was significantly inhibited in both groups. The ACP-hepatocytes showed significantly higher metabolic activity than con-hepatocytes (Figure 6c). For these results, the human iPSC-derived endoderm could further differentiate into hepatocytes with pharmacokinetic functions.

## 4. Discussion

We optimized the method to differentiate human iPSCs into the endoderm to obtain HiEnts and HiHeps with pharmacokinetic function. We found that prolongation of activin A treatment increased the efficiency of cell differentiation. Moreover, pretreatment with 1% DMSO for 24 h before differentiation increased cell differentiation efficiency toward the endoderm and even mature cells for specific human iPSC lines.

Approximately 20 years ago, studies reported that activin A was crucial for mammalian embryonic stem (ES) cells to differentiate into the endoderm [38]. A’Dmour et al. confirmed that activin A induced human ES cell differentiation into the endoderm. Levels of gene and protein expression of endoderm markers were highest on the third day during the 5-days differentiation [31]. The results of A’Dmour et al. are contrary to ours in that differentiated cells from human iPSC (feeder-free) with 7-days activin A treatment showed higher endoderm-specific gene expression levels (Figure 1b). This inconsistency may be due to the different characteristics between ES cells and iPSCs [39] or the different culture conditions, such as on-feeder culture and feeder-free culture, used for pluripotent stem cells. A study reported that differences in iPS maintenance methods (on-feeder culture vs. feeder-free culture) affected hepatocyte differentiation efficiency, and cells maintained under feeder culture conditions exhibited the highest differentiation efficiency [40]. It was suggested that the differentiation method used for human iPSCs cultured under on-feeder conditions is not suitable for human iPSCs cultured under feeder-free conditions. Thus, we aimed to develop a standard endoderm differentiation method for human iPSCs cultured under feeder-free conditions.

Because Wnt signaling is essential for endoderm differentiation, not only activin A but also Wnt−3α are used to induce endoderm from human iPSCs [41]. Moreover, FGF signaling is important for endoderm differentiation because it regulates epithelial-to-mesenchymal transition (EMT) [42]. Phosphatidylinositol 3-kinase (PI3K) antagonizes the ability of ES cells to differentiate in response to activin A [43]. We compared the effects of protocols A, ABF, and ACP on the differentiation of human iPSCs into the endoderm. Based on these reports, we referenced and optimized our protocols to identify the most suitable one for human iPSCs (feeder-free) (Figure 2a). We have reported that differentiated enterocytes from human iPSCs (feeder-free) exhibited intestinal characteristics similar to human normal small intestine using protocol A [27], which suggests the development of HiEnd through protocol A with high purity. However, in this report, rates of endoderm marker-double-positive cells varied in differentiated cells (Figure 2d). Xu et al. reported that activin A together with BMP4 and FGF2 promoted the differentiation of ES cells to the endoderm [44]. Thus, we used a mixture of activin A, BMP4, and FGF2 (ABF protocol) in this study. However, we could not obtain a high purity of the HiEnd using protocol ABF (Figure 2b). By contrast, we found that cells induced using the ACP protocol were of the highest purity and applicable to multiple human iPSC lines.

DMSO was reported to increase the proportion of pluripotent stem cells in the early G1 phase of the cell cycle and activate the retinoblastoma (Rb) protein. This activation of Rb protein happens by increase in the level of hypophosphorylated Rb and decrease in the levels of phosphorylated and hyperphosphorylated Rb [45]. Because Rb is involved in cell proliferation and differentiation, it could promote iPSC differentiation. Studies have reported that pretreatment with low concentrations of DMSO (0.05–2%) has a beneficial effect on differentiation to three germ layers and cells derived from these germ layers [24,45]. We confirmed the promoting effects of 1% DMSO pretreatment on endoderm differentiation using the K cell line based on endodermal and intestinal marker expression. However, for the Windy cell line, no significant benefit was observed. Compared with that for K, expression levels of small intestine-related marker genes were as high in Windy-derived intestinal cells as that in small intestine cells. Consistently, pretreatment with DMSO did not have an inhibitory effect on differentiation. Considering the positive effect of DMSO pretreatment on endoderm differentiation using the Windy cell line (cultured on-feeder) on SOX17 and GATA4 expression (Figure 3b), we hypothesized that the Windy cell line-derived endoderm generated by the ACP protocol reached a high degree of cell differentiation. Thus, DMSO pretreatment would not lead to a significant promotion of differentiation, which warrants further discussion.

We adapted the optimized endodermal differentiation method (protocol ACP) to intestinal and hepatic differentiation. Regarding intestinal differentiation, we confirmed that HiEnts exhibit pharmacokinetic functions of P-gp and CYP3A4, which are closely related to drug absorption, metabolism, and drug–drug interaction using the differentiation method we established (Figure 5a–d). By contrast, ACP-hepatocytes showed higher expression levels of hepatic markers and drug-metabolizing enzymes and higher CYP3A4/5 activity compared to con-hepatocytes (Figure 6a–c). Although we used the same differentiation method from endoderm to hepatocytes in both groups, the properties of the hepatocytes showed different characteristics suggested that the novel endodermal differentiation method led to high efficacy of hepatic differentiation. Based on the evidence, we believe that we succeeded in modifying the endoderm differentiation method.

In this study, we generated enterocytes and hepatocytes with pharmacokinetic functions using our protocol. The differentiated enterocytes or hepatocytes can be employed in drug efficiency- and safety-evaluation models. Moreover, we can set up the small intestine–liver network, which can lead to a more accurate estimation of drugs, using differentiated enterocytes and hepatocytes. Moreover, the evaluation of drugs by these differentiated cells can accelerate the development of personalized medicine. The endoderm can differentiate into other kinds of cells, such as the pancreas and thyroid cells. Further research should be undertaken to expand the application of this protocol and establish disease models.

## 5. Conclusions

In this study, we developed a novel method for differentiating the HiEnd. The HiEnd formed using this method showed endoderm-like homogeneous morphologies, high gene expression levels of endoderm markers, and a high percentage of endoderm marker expressing double-positive cells.

In enterocytes differentiated using this method, gene expression levels of small intestinal markers were similar to those of the human adult small intestine. We also confirmed the presence of tight junctions and small intestinal markers by immunofluorescence staining. Pharmacokinetic functions (P-gp and CYP3A4/5) were confirmed in the enterocytes derived from the modified endoderm.

Furthermore, hepatocytes differentiated from the endoderm showed higher gene expression levels of hepatocyte-related markers and drug-metabolizing enzymes, higher protein expression of hepatocyte-related markers and drug transporters, and higher CYP3A4/5 activity.

Therefore, we can attain a highly pure population of endodermal cells using the ACP protocol. Further, the endoderm can differentiate into enterocytes and hepatocytes, and can used as an in vitro assay model for drug development.

## Figures and Tables

**Table 1:** Real-time PCR primer list.

Gene Name	Sense (5′→3′)	Antisense (5′→3′)
*Oct4*	AGCGAACCAGTATCGAGAAC	TTACAGAACCACACTCGGAC
*SOX17*	TGCAGGCCAGAAGCAGTGTTAC	CCCAAACTGTTCAAGTGGCAGA
*GATA4*	TAGCCTTGTGGGGAGAGCTT	TGGCCTGTCATCTCACTACG
*FOXA1*	AGGCCTGAGTTCATGTTGCT	AGGGCTGGATGGTTGTATTG
*CDX2*	ACCTGTGCGAGTGGATGC	TCCTTTGCTCTTGCGGTTCT
*PAX6*	GCAACATCCGTGGAGAAAAC	AAAAGGCCTCACACATCTGC
*Ki67*	GACTTTGGGTGCGACTTGAC	ACCCCGCTCCTTTTGATAGT
*LGR5*	TGCTCTTCACCAACTGCATC	CTCAGGCTCACCAGATCCTC
*Villin 1*	AGCCAGATCACTGCTGAGGT	TGGACAGGTGTTCCTCCTTC
*P-gp*	CCCATCATTGCAATAGCAGG	TGTTCAAACTTCTGCTCCTGA
*CYP3A4*	CTGTGTGTTTCCAAGAGAAGTTAC	TGCATCATCAATTTCCTCCTGCAG
*ALB*	GAGCTTTTTGAGCAGCTTGG	GGTTCAGGACCACGGATAGA
*BSEP*	TGAGCCTGGTCATCTTGTG	TCCGTAAATATTGGCTTTCTG
*HPRT*	CTTTGCTTTCCTTGGTCAGG	TCAAGGGCATATCCTACAACA

**Table 2:** Primary and secondary antibodies used for immunofluorescence staining.

Antibodies	Source	Dilution
anti-ZO−1	Thermo Fisher Scientific	1:100
anti-villin	Santa Cruz Biotechnology	1:50
anti-Muc2	Santa Cruz Biotechnology	1:100
anti-P-gp	Abcam	1:25
anti-albumin	Abcam	1:100
anti-MRP2	Bioss Antibodies Inc.	1:100
anti-AFP	Santa Cruz Biotechnology, Inc.	1:100
anti-rabbit (Alexa Fluor 488)	Thermo Fisher Scientific	1:200
anti-mouse (Alexa Fluor 568)	Thermo Fisher Scientific	1:200

## Data Availability

The data presented in this study are available on request to the authors.

## Supplementary Materials

The following are available online at https://www.mdpi.com/article/10.3390/cells10040812/s1, Figure S1: Comparison of human iPSC maintenance method (on-feeder and feeder-free) in endoderm differentiation using protocol 72 h activin A, Table S1: List of reagents used for cell differentiation.
