# Supplementary material for: An Efficient Method for the Differentiation of Human iPSC-Derived Endoderm toward Enterocytes and Hepatocytes
Source: Cells. 2021 Apr 6;10(4):812. doi: 10.3390/cells10040812 (PMC8067398; doi:10.3390/cells10040812)
Supplement: Supplementary file 1 [file cells-10-00812-s001.zip › supplementary table s1.pdf]

Table S1. List of reagents used for cell differentiation

Reagent	Source	Catalog number
100 units/mL Penicillin G, 100 µg/mL streptomycin sulfate	Cosmo Bio Co.	03-031-1B
2-Mercaptoethanol	Sigma-Aldrich	M3148-100ML
5-aza-2'-deoxycytidine	Wako Pure Chemical Industries	018-20941
A-83-01	Wako Pure Chemical Industries	517-75771
Activin A	PeproTech Inc.	120-14P
Advanced RPMI1640 medium	Thermo Fisher Scientific	12633012
B27 supplement	Thermo Fisher Scientific	17504001
B27 supplement minus vitamin A	Thermo Fisher Scientific	12587010
Basic fibroblast growth factor	PeproTech Inc.	100-18B
Bone morphogenetic protein-4	R&D systemes, Inc.	Cat# 314-BP
Celecoxib	Tokyo Chemical Industry Co. Ltd	032-24841
CHIR99021	Focus Biomolecules	10-1279
Cosmedium 004	Cosmo Bio Co.	CSR-COS-PM04
Dexamethasone	Wako Pure Chemical Industries	041-18861
DMEM/F-12	Wako Pure Chemical Industries	042-30795
Epidermal growth factor	PeproTech Inc.	AF-100-15
Fetal bovine serum	Sigma-Aldrich	F7524-500ML
GlutaMAX	Thermo Fisher Scientific	35050061
Growth factor reduced Matrigel	BD Biosciences	354230
Hepatocyte growth factor	PeproTech, Inc.	100-39H
iMatrix-511 silk	Matrixome Inc.	892021
KnockOut Serum Replacement	Thermo Fisher Scientific	10828028
L-glutamine	Wako Pure Chemical Industries	BLG-030201B-100ML
Minimum essential medium nonessential amino acid solution	Wako Pure Chemical Industries	ICN- 1681049-100ML
mTeSR1	Veritas Corporation	ST-85850
Oncostatin M	Wako Pure Chemical Industries	158-03413
PD98059	Wako Pure Chemical Industries	169-19211
PI-103	ChemScene LLC	CS-0127
RPMI 1640 + GlutaMAX medium	Thermo Fisher Scientific	61870127
Valproic acid	Wako Pure Chemical Industries	227-01071
Vascular endothelial growth factor	R&D systemes, Inc.	Cat# 293-VE
Y-27632	Focus Biomolecules	10-2301
