# Supplementary material for: An Efficient Method for the Differentiation of Human iPSC-Derived Endoderm toward Enterocytes and Hepatocytes
Source: Cells. 2021 Apr 6;10(4):812. doi: 10.3390/cells10040812 (PMC8067398; doi:10.3390/cells10040812)
Supplement: Supplementary file 1 [file cells-10-00812-s001.zip › supplementary-Figure s1.pdf]

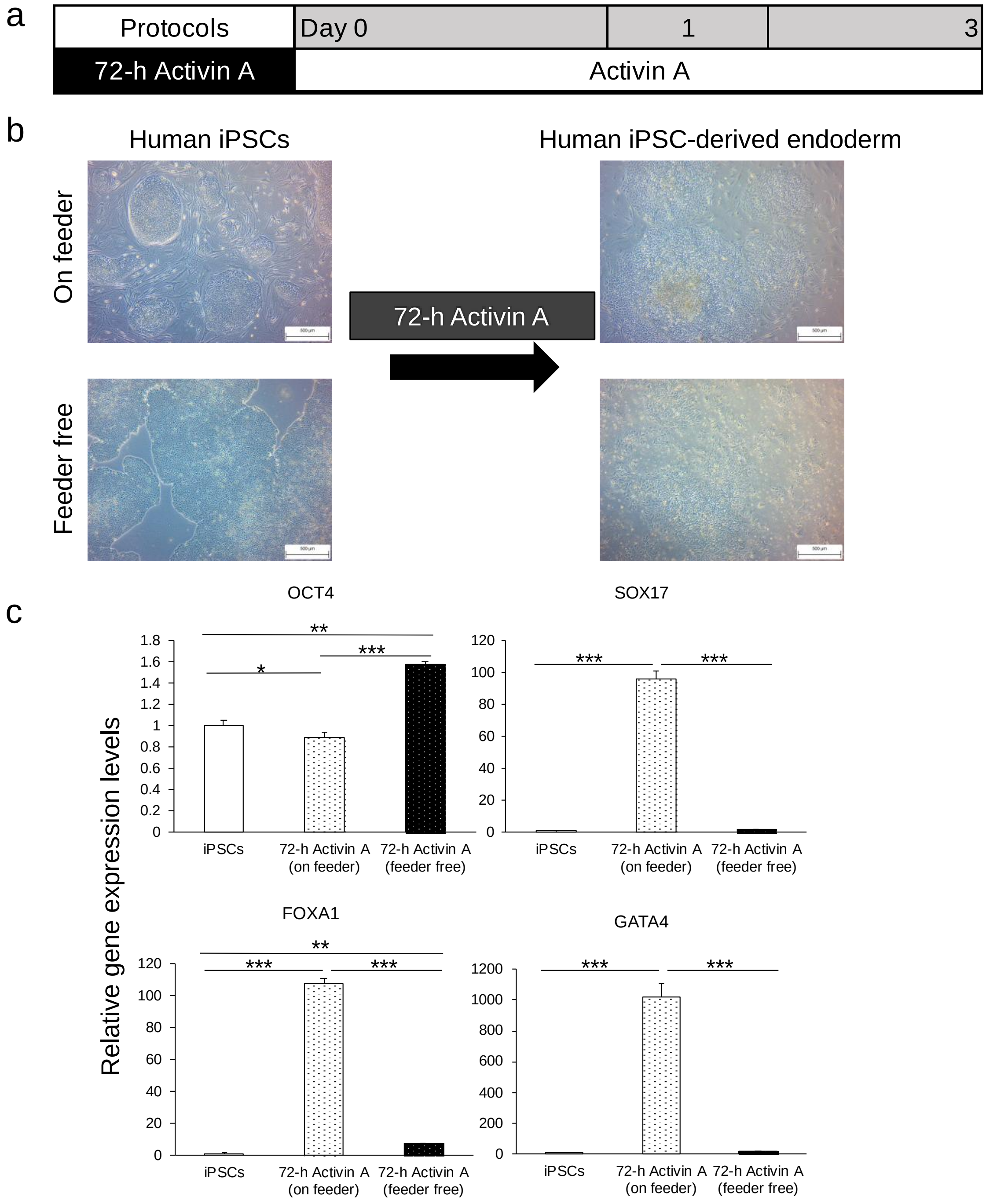

Figure S1. Comparison of human iPSC maintenance method (on feeder and feeder free) in endoderm differentiation using 72-h activin A protocol. (a) Human iPS cell-derived endoderm differentiation method using 72-h activin A protocol. (b) Morphology of the human iPS cell line Windy (on feeder and feeder free) and human iPS cell (Windy)-derived endoderm using 72-h activin A protocol. (c) Relative gene expression levels of OCT4, SOX17, FOXA1, and GATA4. All data are presented as mean ± S.D. (n = 3). Levels of statistical significance: *p < 0.05, **p < 0.01, ***p < 0.001. Scale bar = 500 μm.
